# Associations of Insomnia, Fatigue, and Overtime Work With Self‐Reported Medication Errors Among Perianesthesia Nurses: A Cross‐Sectional Study

**DOI:** 10.1111/inr.70233

**Published:** 2026-09-06

**Authors:** Yulim Kang, Eun Young Choi, Wonhee Baek, Chang Park, Ari Min

**Affiliations:** ^1^ Chung‐Ang University Hospital Seoul South Korea; ^2^ Department of Nursing The Graduate School Chung‐Ang University Seoul South Korea; ^3^ Department of Nursing Chung‐Ang University Seoul South Korea; ^4^ College of Nursing Gyeongsang National University Jinju‐si Gyeongsangnam‐do South Korea; ^5^ College of Nursing University of Illinois Chicago Illinois USA

**Keywords:** fatigue, insomnia, medication error, overtime, patient safety, perianesthesia nursing, shift work

## Abstract

**Aim:**

To examine the associations of insomnia, fatigue, and overtime work with self‐reported medication errors among perianesthesia nurses in South Korea.

**Background:**

Perianesthesia nurses perform high‐risk medication tasks requiring sustained attention, but often experience insomnia, fatigue, and overtime that may impair cognitive performance. Their associations with medication errors remain understudied, particularly in South Korea.

**Design:**

A cross‐sectional survey, reported following the Strengthening the Reporting of Observational Studies in Epidemiology checklist.

**Methods:**

We surveyed 213 perianesthesia nurses across South Korean hospitals. Insomnia and fatigue were measured with validated instruments, and participants reported recent overtime work and medication errors over the previous three months across twelve error types. Negative binomial regression examined associations, adjusting for demographic and work‐related characteristics.

**Results:**

Clinically significant insomnia was present in one‐quarter of nurses, fatigue in two‐thirds, and recent overtime in four in five. In adjusted analysis, insomnia and fatigue were significantly associated with higher summed self‐reported medication‐error‐type frequency scores, whereas overtime work was not. Estimates remained directionally consistent across sensitivity analyses, although the insomnia association was not significant when the outcome was dichotomized.

**Conclusion:**

Clinically significant insomnia and fatigue were associated with a higher summed self‐reported medication‐error‐type frequency score, whereas an association with overtime work was not supported. These potentially modifiable factors warrant further prospective investigation to determine whether addressing them improves medication safety in high‐acuity perianesthesia settings.

**Implications for Nursing:**

Nurse managers may consider evaluating and implementing system‐level strategies to monitor and mitigate fatigue and sleep disturbances among perianesthesia nurses to support medication safety and nurses’ well‐being.

**Implications for Health Policy:**

Policymakers could support these organizational efforts by incorporating fatigue management into workforce and patient safety standards.

## 1 Introduction

Patient safety is a fundamental goal of healthcare (World Health Organization 2009). Since the *To Err Is Human* report (National Academy of Medicine 2000), reducing preventable harm has become a core value of health systems worldwide. Despite 25 years of progress in safety culture and reporting systems, medication errors, particularly those committed by nurses during medication administration, remain a leading cause of preventable harm and are associated with workforce factors such as long work hours and fatigue (Bates and Singh 2018). Medication administration is among the most frequent yet high‐risk nursing tasks. Extended work hours and overtime are associated with increased reporting of adverse outcomes, including medication errors, in nursing populations (Rogers et al. 2004). In South Korea, approximately 80% of hospital nurses report overtime, with some exceeding 8 additional hours weekly (Korean Hospital Nurses Association 2023).

Long work hours and shift work can reduce sleep duration and disrupt sleep quality, both of which may contribute to fatigue and impair attention, reaction time, and clinical performance (Bae and Fabry 2014). Conceptually distinct from short‐term sleep deprivation, insomnia is a chronic condition characterized by persistent difficulty initiating or maintaining sleep despite adequate sleep opportunity (Sateia 2014). In a Korean study, 38.7% of shift‐working nurses reported insomnia symptoms, which were significantly associated with longer night shifts and extended weekly work hours (Shin and Kim 2020). Although sleep disturbances are common across shift‐working industries — a multicenter Korean study of over 33,000 shift workers reported an insomnia prevalence of 38.7% (Sim et al. 2021) — the burden appears more pronounced in nursing, as rapid rotation and high psychological distress compound sleep fragmentation (Booker et al. 2018; Shin and Kim 2020).

Workplace fatigue in nursing is a multidimensional phenomenon arising from sustained cognitive and emotional demand, prolonged hours, and circadian disruption, in addition to insufficient sleep (Caruso 2014; Smith‐Miller et al. 2014). Sleep deprivation and fatigue are well‐established predictors of safety incidents in healthcare settings (Stimpfel and Aiken 2013). The perianesthesia environment intensifies these risks; unlike general wards, it presents acute, unpredictable surgical extensions and emergence episodes, producing intensive fatigue spikes that differ qualitatively from cumulative ward burnout (Min et al. 2024). Sleep loss and accumulated fatigue impair attention, psychomotor speed, working memory, and executive function (Lim and Dinges 2010): the very resources required for accurate medication preparation, multistep verification (e.g., the “five rights” of medication administration), and error detection. Consistent with this, US nurses working shifts of ≥12 h showed approximately a threefold higher risk of self‐reported errors (Rogers et al. 2004).

These risks are critical in anesthesia care, where drug administration, hemodynamic management, and monitoring occur within narrow time margins and with limited recovery from error. Perianesthesia nurses administer anesthetics and sustain respiratory and circulatory stability perioperatively (Rayborn and Jeong 2024). In a national analysis of Korea's voluntary patient safety reporting system, operating and recovery units accounted for only 4.6% of reported incidents in hospitals with ≥500 beds, a low proportion likely reflecting the brief patient stay in these settings; however, incidents in these hospitals were about 2.5 times more likely to reach adverse‐event severity, reflecting the high‐risk nature of perianesthesia care (Kim 2020).

In Korea, the practice scope of perianesthesia nursing spans anesthesia induction through post‐anesthesia recovery yet often lacks formal role standardization (Jeon et al. 2017), and studies of its working conditions and medication safety remain scarce. Over 60% of the tasks of perianesthesia nurses involve multitasking, such as preparing medications while monitoring patients (Yoo et al. 2021). During such high‐demand episodes, working memory and attentional load can exceed available capacity, producing slips and lapses (Reason 2000); these risks are amplified when capacity is already constrained by sleep loss or fatigue.

Overtime is a further concern: surgical unpredictability often pushes perianesthesia nurses beyond scheduled hours (Battié et al. 2017). Beyond same‐shift fatigue, overtime reduces restorative sleep before the next shift, contributing to cumulative sleep debt and chronic sleep restriction (Caruso 2014). Excessive hours have been linked to missed care and clinical errors (Wu et al. 2018), yet overtime in Korean perianesthesia settings remains understudied.

International research has examined medication errors in anesthesia care (Bratch and Pandit 2021); however, Korean perianesthesia nurses have been largely excluded, and their practice operates without formal role standardization or certification and under less regulated work‐hour conditions; therefore, whether existing evidence generalizes to this setting remains unclear. No study has examined the associations of insomnia, fatigue, and overtime with the frequency of self‐reported medication errors in this population. This study, therefore, aimed to (1) assess the prevalence of insomnia, fatigue, and overtime among Korean perianesthesia nurses and (2) examine their associations with the frequency of self‐reported medication errors.

## 2 Methods

### 2.1 Research Design

This cross‐sectional, descriptive correlational study examined the associations of insomnia, fatigue, and overtime work with the frequency of self‐reported medication errors among hospital perianesthesia nurses. It is reported in accordance with the STROBE guideline for cross‐sectional studies.

### 2.2 Sample and Setting

Participants were perianesthesia nurses actively providing anesthesia care at tertiary, general, or specialized hospitals in South Korea, with adequate communication and literacy and voluntary consent. Nurses not directly involved in perianesthesia care (e.g., managers, outpatient nurses), those with <3 months of clinical experience, and those working exclusively in recovery rooms were excluded. Using G*Power 3.1.9.7 (a Poisson approximation for negative binomial regression, which G*Power does not directly support), a medium effect (incidence rate ratio [IRR] = 1.35–1.65), α = .05, and power = .95 yielded a required sample of 77 (IRR = 1.65) to 230 (IRR = 1.35).

### 2.3 Measures

#### 2.3.1 Insomnia

Insomnia was assessed with the Korean Insomnia Severity Index (ISI‐K; Morin 1993; Cho et al. 2014): seven items rating insomnia symptoms over the past 2 weeks on a 0–4 scale (total 0–28; ≥15 = clinically significant insomnia), including three items on sleep‐related problems and others on sleep satisfaction, daytime functioning, quality of life impairment, and sleep‐related distress. The ISI‐K has demonstrated strong psychometric properties in Korean populations (Cho et al. 2014), with Cronbach's α reported as 0.92. In this study, the reliability coefficient was 0.85.

#### 2.3.2 Fatigue

Fatigue was assessed with the Korean Chalder Fatigue Questionnaire (CFQ‐K; Chalder et al. 1993; Ha et al. 2018): 11 items on symptoms experienced over the past month, rated on a 4‐point Likert scale. Binary scoring was applied; responses of “less than usual” or “no more than usual” were scored as 0, whereas those of “more than usual” or “much more than usual” were scored as 1 (total 0–11; ≥4 = fatigue). In addition to the binary score, a total Likert score (with each item scored 0 to 3; range 0–33) was computed to summarize the overall fatigue severity and enable comparison with prior studies that reported Likert‐scored means. The binary score was used for all classification and inferential analyses; the Likert score was reported descriptively only. The reliability at the time of the instrument's development was reported as a Cronbach's α value of 0.89 (Chalder et al. 1993), and comparable reliability (Cronbach's α = 0.88) was reported for the Korean version (Ha et al. 2018). The CFQ‐K also demonstrated good factorial and concurrent validity (Ha et al. 2018). Cronbach's α in this study was 0.87 for the binary score and 0.91 for the total Likert score.

#### 2.3.3 Overtime

Overtime was self‐reported via an open‐ended item for both the past week (primary measure) and past month (used in sensitivity analyses), recorded in 30‐min increments. The 1‐week window was selected as the exposure most proximal to current insomnia, fatigue, and cognitive states relevant to medication errors and because shorter reference periods support more accurate recall (Stull et al. 2009). Responses were dichotomized as “any” versus “no overtime”; this way, the regression coefficient could reflect the relative expected value of the summed self‐reported medication‐error‐type frequency score for nurses with versus without recent overtime.. Sensitivity analyses further modeled continuous overtime hours under both reference periods.

#### 2.3.4 Self‐Reported Medication Errors

Self‐reported medication errors were defined as errors the participant committed during medication administration within the past 3 months, regardless of formal reporting; near‐misses and harm severity were not separately captured. A 12‐item scale measured the frequency of each error type over this period on a 6‐point scale (0 = never, 5 = ≥5 times), which was summed to a total score, hereafter referred to as the summed self‐reported medication‐error‐type frequency score. Because more than one error type may have occurred within a single incident, this score does not necessarily represent the number of distinct medication‐error incidents. The 3‐month recall period balanced capturing enough of these relatively infrequent events with maintaining acceptable recall accuracy, as discrete errors are recalled more reliably than routine activities. Items were adapted from Hicks et al. (2007), based on the US Pharmacopeia's error classification (e.g., omission, wrong time, improper dose, and prescribing errors). In this study, a prescribing error refers to instances where a physician's erroneous order was not identified by the nurse, and the medication was administered as prescribed; it did not include transcription errors or nurse‐initiated changes to a prescription order. The scale was developed for this study through cognitive interviews with five perianesthesia nurses and expert review by four senior nurses and four nursing professors, after which item wording was refined, clinical examples were added, and an “other errors” category was included to capture events such as expired‐medication use or air bubbles during infusion. The resulting item‐level content validity index (CVI) was 0.90–1.00, scale‐level CVI was 0.97, and Cronbach's α was 0.86.

#### 2.3.5 General and Work‐Related Characteristics

Demographic variables (age [continuous, years], sex, marital and parental status, education, caffeine and alcohol use) and work‐related variables (perianesthesia experience [continuous, months], shift type, scope of practice, hospital type, number of beds, commuting time, monthly night shifts, and absenteeism, presenteeism, and productivity loss) were collected as potential confounders, with categorical variables using predefined options. Caffeine and alcohol were included because of their effects on sleep and alertness (Clark and Landolt 2017; Gardiner et al. 2025). Shift type was classified as fixed or rotating. A fixed schedule was nonrotating and worked predominantly during the day, whereas a rotating schedule cycled through either a three‐shift (8‐h day, evening, and night shifts) or a two‐shift system (12‐h day and night shifts). Scope of practice was classified as anesthesia only or anesthesia combined with recovery care. Commuting time (five options) was collapsed into ≤30, 31–60, and ≥61 min. Night shifts were counted over the past month to match the monthly rotation cycle, providing a more stable estimate than a weekly count. Absenteeism (occurrence and days), presenteeism (occurrence and days), and productivity loss (0–10 scale) were each assessed for the past month.

### 2.4 Data Collection

Data were collected March 26–28, 2025, until the target sample size was reached. Nurses were recruited through online nursing communities, shift‐management applications, and social media, supplemented by snowball sampling; eligibility was screened using two filtering questions before electronic consent and a 10–15‐min survey. Because the filtering questions screened eligibility at the level of work setting, respondents who passed the filters but whose survey responses indicated that they did not perform anesthesia nursing duties were identified and excluded during data cleaning. Because recruitment used multiple open‐access channels and snowball sampling, the number of eligible nurses exposed to the materials (and thus a precise response rate) could not be ascertained.

All survey items required a response before submission, so only completed questionnaires could be submitted, and no missing data occurred for any study variable. Submitted questionnaires were screened for careless responding (straight‐line responding across all items) and for duplicate participation. Screening for duplicate participation involved checking entries against the list of mobile telephone numbers collected for delivery of the coffee‐voucher incentive.

### 2.5 Data Analysis

Analyses were performed using Stata 19 (StataCorp, College Station, TX, USA). Because the summed self‐reported medication‐error‐type frequency score comprises nonnegative integer values with a right‐skewed, nonnormal distribution (Shapiro–Wilk *W* = 0.82, *p* < .001; skewness = 1.98), Mann–Whitney *U* tests were used for bivariate comparisons across insomnia, fatigue, and overtime status; negative binomial regression was used for multivariable analysis, as this model is appropriate for nonnegative integer outcomes with skewed distributions (Cameron and Trivedi 2013; Hilbe 2011). The choice of negative binomial over Poisson regression was based on evidence of substantial overdispersion in a Poisson model fitted for comparison (Pearson chi‐square/*df* = 4.46; likelihood‐ratio test of α = 0, *p* < .001), which underestimates standard errors and inflates Type I error risk if unaddressed. A zero‐inflated specification did not improve fit (NB AIC = 940.4; ZINB AIC = 942.4); therefore, the standard negative binomial model was retained. Exponentiated coefficients from the negative binomial model are reported as IRRs; because the outcome is a summed self‐reported medication‐error‐type frequency score, each IRR reflects the relative expected value of this score and should not be interpreted as the incidence rate of distinct medication‐error events.

All three exposures were entered as binary variables: clinically significant insomnia (versus none), fatigue defined by the binary CFQ scoring (score of 4 or higher), and any overtime work in the past week (versus none); the Likert‐scored CFQ total was used for descriptive purposes only. Covariates were specified a priori following the disjunctive cause criterion (VanderWeele 2019): a variable was eligible for adjustment if prior evidence indicated that it is a determinant of medication errors, insomnia or fatigue, or both. On this basis, the model was adjusted for demographic and family factors (age, sex, marital status, and children), which have been examined in relation to sleep disturbance and fatigue in shift‐working and nursing populations (Alahmadi and Alharbi 2018; Booker et al. 2018; Brito et al. 2021); education and clinical background (educational level, perianesthesia experience, and scope of practice), examined in relation to medication errors and error‐related practice (Björkstén et al. 2016; Chang and Mark 2009; Kerari and Innab 2021; Özen et al. 2019); work‐structure factors (type of shift work, number of night shifts, number of beds, commuting time, and presenteeism), examined in relation to fatigue and medication errors (Daouda et al. 2022; Di Muzio et al. 2019; Flynn et al. 2012; Letvak et al. 2012; Saleh et al. 2014); and lifestyle factors (caffeine intake frequency and alcohol intake per consumption), examined in relation to sleep quantity and quality (Clark and Landolt 2017). Hospital type, absenteeism, and the productivity‐loss score were designated in advance as alternates and not entered into the final model, as hospital type overlaps conceptually with the number of beds, and absenteeism and productivity loss overlap with presenteeism.

As a sensitivity analysis, binary logistic regression compared nurses reporting at least one error type with those reporting none, using the same covariates as the principal model. Item‐level comparisons across the 12 error types and three exposures (36 tests) were treated as exploratory, with the Benjamini–Hochberg false discovery rate procedure (*q* = .05) applied to control for multiple testing. For all other analyses, statistical significance was set at *p* < .05.

### 2.6 Ethical Considerations

The study was approved by the Chung‐Ang University Institutional Review Board (Approval no. 1041078‐20241206‐HR‐349). Participants gave electronic informed consent; confidentiality and the right to withdraw were ensured, and mobile telephone numbers collected solely for incentive delivery and duplicate screening were stored separately from survey responses and deleted after use.

## 3 Results

### 3.1 Characteristics of the Sample

Of the 237 nurses who responded, 24 were excluded (*n* = 23 worked in operating or post‐anesthesia care areas but did not perform anesthesia nursing duties; *n* = 1 provided careless responses), and no duplicate entries were identified. The remaining 213 nurses met the inclusion criteria and were included in the analysis (Table 1).

**TABLE 1:** General characteristics of the nurses (*N* = 213).

	*n*	%	Mean	*SD*
Age (years)			30.34	4.31
Sex				
Male	16	7.5		
Female	197	92.5		
Marital status				
Unmarried	165	77.5		
Married	48	22.5		
Children				
Yes	32	15.0		
No	181	85.0		
Education level				
Associate degree	18	8.5		
Bachelor's degree	185	86.9		
Master's degree or higher	10	4.7		
Caffeine intake frequency				
Do not consume caffeine	25	11.7		
1–2 cups per month	2	0.9		
1–2 cups per week	31	14.6		
1–2 cups per day	114	53.5		
Consume 3 or more cups per day	41	19.2		
Alcohol intake frequency				
Do not consume alcohol	23	10.8		
<1 time per month	50	23.5		
1 time per month	44	20.7		
2–4 times per month	72	33.8		
2–3 times per week	18	8.5		
≥4 times per week	6	2.8		
Alcohol intake per consumption (*n* = 190)				
1–2 drinks	72	37.9		
3–4 drinks	37	19.5		
5–6 drinks	40	21.1		
≥7 drinks	41	21.6		
Working experience of perianesthesia nurse (years)				
<1	32	15.0		
≥1, <5	143	67.1		
≥5	38	17.8		
Type of shift work				
Fixed shift	74	34.7		
Rotating shift	139	65.3		
Scope of practice				
Anesthesia nursing	92	43.2		
Anesthesia and recovery care	121	56.8		
Hospital type				
Tertiary hospital	96	45.1		
General and other hospitals	117	54.9		
Number of beds				
≥1000	50	23.5		
500–999	101	47.4		
300–499	39	18.3		
<300	23	10.8		
Commuting time (min)				
≤30	109	51.2		
31–60	72	33.8		
≥61	32	15.0		
Night shift in the last month				
Yes	160	75.1		
No	53	24.9		
Number of night shifts in the last month			3.47	2.60
0–3	106	49.8		
4–6	87	40.8		
≥7	20	9.4		
Experience of absence in the last month				
Yes	12	5.6		
No	201	94.4		
Number absent from work due to health problems (*n* = 12) ^*^			1.92	1.16
Experience of presenteeism in the last month				
Yes	118	55.4		
No	47	22.1		
Not sick	48	22.5		
Number of days worked while being sick (*n* = 118)^*^			2.52	1.81
Level of reduced work productivity (0–10) (*n* = 118)^*^			5.06	2.18

*Note*: SD = standard deviation.

* Only nurses who answered that they experienced presenteeism or absenteeism.

Most were female (92.5%), unmarried (77.5%), and held a bachelor's degree (86.9%), with a mean age of 30.34 ± 4.31 years; 88.3% consumed caffeine, and 89.2% reported alcohol use. Most had 1–5 years of perianesthesia experience (67.1%), worked rotating shifts (65.3%), and performed combined anesthesia and recovery care (56.8%); 45.1% worked at tertiary hospitals. Three‐quarters (75.1%) had worked night shifts in the past month (mean 3.47 ± 2.60). Additionally, in the past month, 5.6% had been absent for health reasons (mean 1.92 ± 1.16 days), and 55.4% reported presenteeism (mean 2.52 ± 1.81 days; productivity‐loss score 5.06 ± 2.18 on a 0–10 scale).

### 3.2 Frequency and Types of Self‐Reported Medication Errors

**FIGURE 1:**
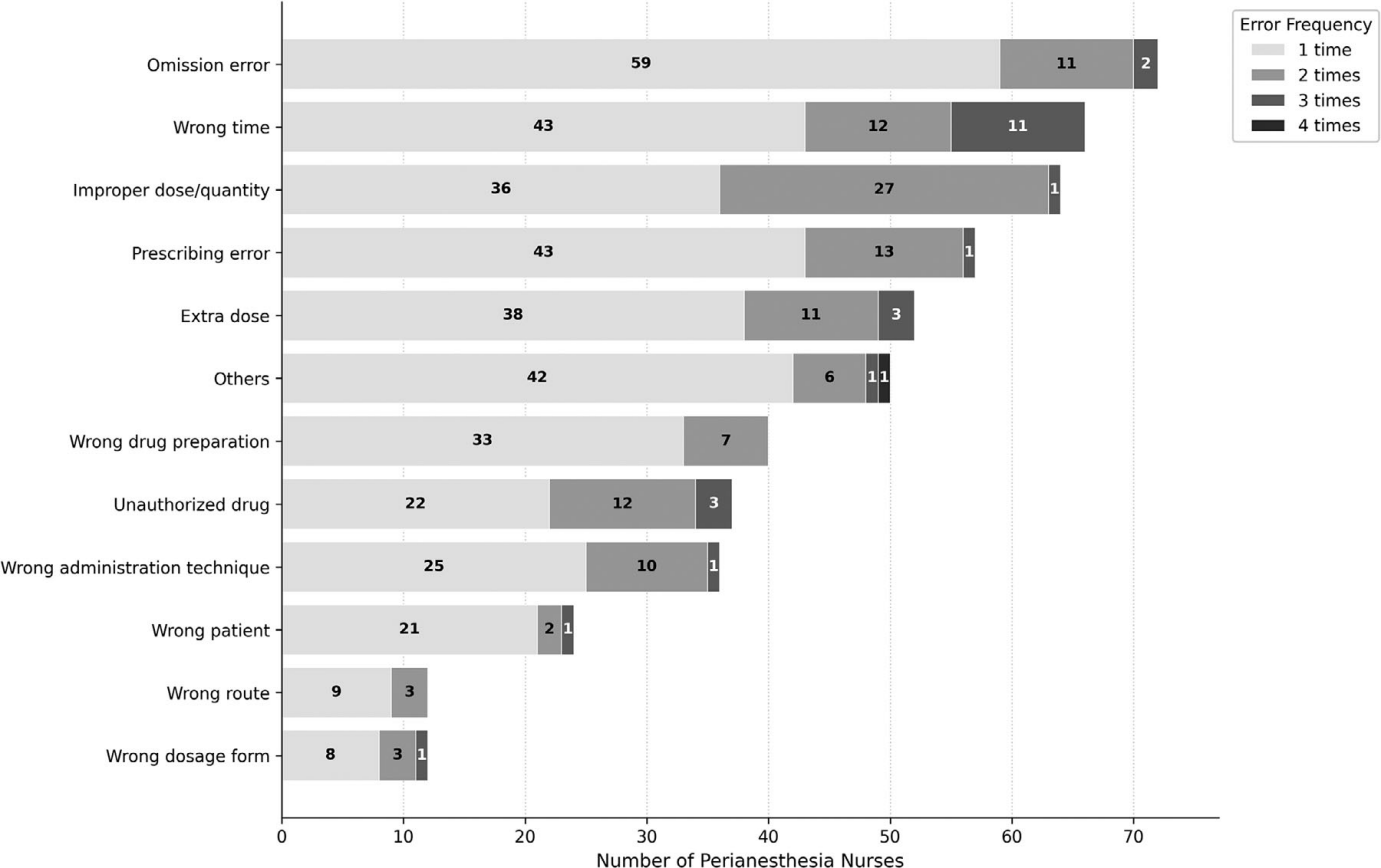
Distribution of the reported frequency of each self‐reported medication error type among the 140 perianesthesia nurses who reported at least one medication error type (of 213 participants). Each bar represents the number of nurses who reported a specific error type, segmented by how often that type occurred (1–4 times), and the numbers within the bars indicate how many nurses reported each frequency. Although “five or more times” was an available response option, no nurse selected it. Each nurse could report multiple error types; therefore, the categories are not mutually exclusive. This figure therefore shows frequencies at the item level, whereas the summed self‐reported medication‐error‐type frequency score in Table 2 is the nurse‐level total across all 12 items.

Over the past 3 months, nurses reported a mean summed self‐reported medication‐error‐type frequency score of 3.25 ± 4.36, with the variance (19.0) far exceeding the mean, indicating overdispersion (Table 2). A third (34.3%) reported no errors, whereas 9.4% reported 10 or more errors. Among the 140 nurses reporting errors (Figure 1), the most common types were omission (51.4%), wrong time (47.1%), improper dose/quantity (45.7%), and prescribing errors (40.7%); the least common were wrong route and wrong dosage form (8.6% each).

**TABLE 2:** Distribution of the summed self‐reported medication‐error‐type frequency score among perianesthesia nurses (*N* = 213).

	*n*	%	Mean	*SD*
Sum score			3.25	4.36
0	73	34.3		
1	37	17.4		
2	19	8.9		
3	15	7.0		
4	9	4.2		
5	10	4.7		
6	11	5.2		
7	10	4.7		
8	4	1.9		
9	5	2.3		
≥10	20	9.4		

*Note*: SD = standard deviation. Because a single incident can involve more than one error type, the summed score may exceed the number of distinct medication‐error incidents.

### 3.3 Prevalence of Insomnia, Fatigue, and Overtime Work and Their Association With the Summed Self‐Reported Medication‐Error‐Type Frequency Score

Insomnia, fatigue, and overtime levels and corresponding self‐reported error frequencies are summarized in Table 3. Clinically significant insomnia was present in 26.8% of the participants (mean ISI 10.98 ± 5.21), fatigue in 67.6% (binary CFQ ≥4; mean Likert‐scored CFQ total 16.38 ± 6.44, observed range 0–32), and recent overtime in 80.3% (mean 2.22 ± 1.95 h). The summed self‐reported medication‐error‐type frequency score differed significantly by all three: insomnia (median 4.0 [IQR 2.0–9.0] vs. 1.0 [0.0–3.0]; *U* = 2425.50, *p* < .001), fatigue (2.0 [1.0–6.0] vs. 0.0 [0.0–2.0]; *U* = 2921.50, *p* < .001), and overtime (2.0 [0.0–6.0] vs. 0.0 [0.0–1.75]; *U* = 2361.50, *p* < .001).

**TABLE 3:** Summed self‐reported medication‐error‐type frequency score according to insomnia, fatigue, and overtime work (*N* = 213).

	*n* (%)	Median [IQR]	Mann–Whitney *U*	*p‐*value
Insomnia				
Absence of insomnia	156 (73.2)	1.0 [0.0–3.0]	2425.50	<.001
Clinically significant insomnia	57 (26.8)	4.0 [2.0–9.0]		
Fatigue				
Nonfatigued	69 (32.4)	0.0 [0.0–2.0]	2921.50	<.001
Fatigued	144 (67.6)	2.0 [1.0–6.0]		
Overtime work				
Yes	171 (80.3)	2.0 [0.0–6.0]	2361.50	<.001
No	42 (19.7)	0.0 [0.0–1.75]		

*Note*: IQR = interquartile range. The Mann–Whitney *U* test was performed for all comparisons. Fatigued defined by binary CFQ scoring (≥4).

To explore these associations across individual error types, item‐level Mann–Whitney *U* tests were performed as exploratory analyses, with the Benjamini–Hochberg correction applied across the 36 comparisons (Supplementary Table 1). Of the 18 nominally significant associations, 14 remained significant after correction. Insomnia was associated with higher frequencies of five error types (omission, improper dose/quantity, prescribing error, extra dose, and unauthorized drug). Fatigue was associated with these five error types and wrong time and wrong drug preparation. Overtime work was associated with omission and improper dose/quantity. Four nominally significant associations did not survive correction: insomnia with wrong time (*q* = .065) and wrong drug preparation (*q* = .075) and overtime work with wrong drug preparation (*q* = .065) and unauthorized drug (*q* = .084). None of the three exposures was associated with wrong administration technique, wrong patient, wrong route, wrong dosage form, or other errors. Given the exploratory nature of these comparisons, the item‐level findings are interpreted with caution.

### 3.4 Factors Associated With the Summed Self‐Reported Medication‐Error‐Type Frequency Score

In the negative binomial regression adjusting for all covariates, the overall model was significant (LR χ^2^(24) = 81.27, *p* < .001) (Table 4 and Supplementary Table 2). Clinically significant insomnia (β = 0.507, 95% CI 0.057–0.957, *p* = .027; IRR = 1.66) and fatigue (binary CFQ scoring, score of 4 or higher; β = 0.497, 95% CI 0.039–0.955, *p* = .034; IRR = 1.64) were each associated with a higher summed self‐reported medication‐error‐type frequency score. Overtime work showed a positive but nonsignificant association (β = 0.416, 95% CI −0.090 to 0.922, *p* = .107).

**TABLE 4:** Factors associated with the summed self‐reported medication‐error‐type frequency score using negative binomial regression (*N* = 213).

	Summed self‐reported medication‐error‐type frequency score				
			95% CI		
Variables	Regression coefficient (*β*)	Incidence rate ratio	LLCI	ULCI	*p*‐value
Insomnia					
Clinically significant insomnia	0.507	1.66	1.059	2.603	.027
Fatigue					
Fatigued	0.497	1.64	1.040	2.599	.034
Overtime work					
Yes	0.416	1.52	0.914	2.513	.107

*Note*: Reference groups for independent variables: Insomnia = absence of insomnia; Fatigue = nonfatigued (fatigued defined as binary CFQ ≥4); Overtime work in the last week = no. The regression model was adjusted for age, sex, marital status, children, educational level, perianesthesia experience, type of shift work, scope of practice, number of beds, commuting time, number of night shifts, experience of presenteeism, caffeine intake frequency, and alcohol intake per consumption. Full model estimates are presented in Supplementary Table 2. IRR = incidence rate ratio, representing the relative expected value of the summed self‐reported medication‐error‐type frequency score rather than the incidence rate of distinct medication‐error events. LLCI = lower limit of the 95% confidence interval; ULCI = upper limit of the 95% confidence interval.

Two sets of sensitivity analyses supported the robustness of these findings. First, when overtime was operationalized as continuous hours rather than a binary indicator, the pattern was unchanged: overtime remained nonsignificant under both reference periods (weekly: IRR = 1.08 per hour, 95% CI 0.99–1.19, *p* = .095; monthly: IRR = 1.01 per h, 95% CI 0.97–1.04, *p* = .718), whereas insomnia (IRR = 1.64 and 1.67) and fatigue (IRR = 1.71 and 1.73) remained significant and of comparable magnitude. Second, in the binary logistic model comparing nurses reporting at least one error type with those reporting none, fatigue remained significantly associated with the outcome (OR = 2.49, 95% CI 1.07–5.80), whereas insomnia (OR = 2.58, 95% CI 0.86–7.73) and overtime work (OR = 2.05, 95% CI 0.81–5.17) were directionally consistent but nonsignificant, a pattern expected given the reduced statistical power of a dichotomized outcome (Supplementary Table 3).

## 4 Discussion

This study examined the associations of insomnia, fatigue, and overtime work with the frequency of self‐reported medication errors among Korean perianesthesia nurses. Clinically significant insomnia and fatigue were independently associated with a higher summed self‐reported medication‐error‐type frequency score, whereas overtime work was not significantly associated. These associations are particularly consequential in perianesthesia care, where medication administration is often performed independently under time pressure, and errors can lead to failed anesthesia, severe complications, or death (Prabhakar et al. 2015). As potentially modifiable factors, insomnia and fatigue warrant further prospective investigation to determine whether targeted individual and organizational strategies can improve medication safety in this setting.

Approximately 27% of nurses had clinically significant insomnia, 68% reported fatigue, and over 80% reported recent overtime, levels broadly consistent with those in prior nursing studies (e.g., 30.3% insomnia among Korean ICU nurses [Mun and Choi 2020]; a mean Chalder fatigue Likert score of 18.36 among 14,839 nurses [Jang et al. 2021] compared to 16.38 in our sample; and 76.4% of nurses working overtime on their most recent shift [Cho et al. 2016]). However, differences in instruments, cutoff criteria, reference periods, and clinical settings preclude precise quantitative comparison; in particular, mean scores do not directly reflect the proportion of participants experiencing clinically significant symptoms.

Medication errors are an inherent risk in healthcare, and their measured frequency varies with definition and method. Prospective observation studies report errors in approximately 8%–14% of administrations (Jessurun et al. 2023), whereas nurse self‐report yields higher figures: about 54% in a meta‐analysis (Fathizadeh et al. 2024) and 68%–72% in cross‐sectional surveys (Wondmieneh et al. 2020). The proportion of nurses who reported at least one medication error type (65.7%; mean summed score = 3.25 over 3 months) falls within this self‐reported range, likely reflecting capture of minor or unreported errors and our item‐by‐item assessment of 12 error types. The predominance of nurses with limited perianesthesia experience (67.1% with 1–5 years) may have further contributed, given the relationship between a lower experience level and higher error frequency (Cooper et al. 2012). The most frequently reported error types (omission, wrong‐time administration, and improper dosing) are consistent with the findings of Hicks et al. (2007) and typically arise from interacting human, task, and environmental factors. The prescribing error item warrants clarification. Because Korean nurses do not hold independent prescribing authority, this item captured instances in which an erroneous physician order was not identified, and the medication was administered as prescribed, rather than independent prescribing by nurses. As the scope of nursing practice regarding medication orders varies across countries, this finding should be interpreted within the Korean delegation framework. Finally, because our self‐report captured any committed error regardless of formal reporting and allowed multiple error types per incident, direct comparison with voluntary national reporting systems, which capture mainly higher‐severity events, is limited (Korea Institute for Healthcare Accreditation 2024; Koo 2021).

The association between insomnia and the summed self‐reported medication‐error‐type frequency score aligns with previous findings (Mun and Choi 2020). Persistent insomnia reduces sleep duration and continuity, which can worsen fatigue and impair sustained attention, which are plausible pathways to medication errors (Lim and Dinges 2010), although our cross‐sectional design precludes causal inference. In a sensitivity analysis using a dichotomized outcome, the insomnia estimate was directionally consistent and of similar magnitude but did not reach significance, likely reflecting the loss of information when the outcome is dichotomized; this finding is therefore interpreted with corresponding caution. Caffeine intake and night‐shift frequency are known correlates of insomnia in shift workers (Booker et al. 2018) and are common targets of sleep hygiene education. However, such education has shown limited efficacy for established insomnia in shift workers, and schedule modification is constrained in 24‐h care. Therefore, addressing insomnia in this population may require a multicomponent approach including cognitive behavioral therapy for insomnia (CBT‐I) and organizational measures.

Fatigue (binary scoring, CFQ ≥4) was likewise associated with a higher summed self‐reported medication‐error‐type frequency score (IRR = 1.64). Importantly, the CFQ captures general workplace fatigue rather than insomnia‐related fatigue alone: the two are conceptually distinct, with insomnia‐related fatigue arising mainly from chronic sleep difficulty, whereas workplace fatigue reflects the cumulative cognitive, emotional, and physical demands of nursing, of which inadequate sleep is a contributor (Caruso 2014; Smith‐Miller et al. 2014). Consistent with this, fatigue was associated with multiple work and lifestyle variables (shift type, scope, hospital type, commuting, presenteeism, caffeine; data not shown). Direct empirical support comes from wearable‐device data linking self‐reported fatigue to higher rates of medication error (Farag et al. 2024) and from a review in which 31 of 38 studies identified fatigue as a key risk factor for medication errors (Bell et al. 2023). The independent associations of both insomnia and workplace fatigue with medication errors suggest interventions may need to address sleep‐specific and broader work‐environment contributors alike.

Overtime work was not significantly associated with the summed self‐reported medication‐error‐type frequency score in either the binary or continuous specification, despite a univariate difference. This null result is consistent with that of Bae and Fabry (2014), who reported inconsistent overtime effects on patient outcomes. Although the sample exceeded the a priori target, the limited variability in weekly overtime (mean = 2.22, SD = 1.95 h), significantly below the 10.29 h reported for Korean ward nurses (Min et al. 2022), likely reflecting the short but intensive nature of perianesthesia work, may have constrained statistical power. Our data, therefore, did not support an overtime–error association in this setting. Future studies should examine overtime intensity, recovery, and potential interaction effects with insomnia and fatigue to determine whether overtime modifies medication error risk in high‐risk subgroups.

Finally, our findings should be interpreted within the Korean context. Healthcare systems such as the UK's National Health Service (Pickup et al. 2025) have started to adopt fatigue risk management, encompassing scheduling practices, adequate staffing, protected rest, workload monitoring, and evidence‐based sleep interventions (Querstret et al. 2020). However, South Korea has no comparable national framework for nursing; staffing is governed mainly through ratio‐based reimbursement tiers (Kim et al. 2024), and fatigue is seldom treated as a distinct institutional safety risk. Therefore, developing context‐specific strategies adapted to rapid shift rotations and limited rest infrastructure warrants consideration.

### 4.1 Implications for Nursing Management and Nursing Policy

Because insomnia and fatigue cannot be eliminated in a 24‐h care workforce, they are likely to require ongoing system‐based management rather than one‐off fixes. Our findings indicate that insomnia and fatigue were associated with a higher summed self‐reported medication‐error‐type frequency score in this cross‐sectional study. Nurse managers may consider assessing and addressing these two potentially modifiable factors, while treating overtime as part of broader work‐environment monitoring, for example, by tracking fatigue and overtime data within staffing systems (Caruso 2014), rather than as a proven driver of medication error.

Several directions are suggested by the wider literature, although their effects on medication errors remain to be evaluated in this setting. Scheduling practices that limit consecutive shifts and protect inter‐shift recovery are associated with reduced fatigue (Querstret et al. 2020; Qtait et al. 2025); however, circadian‐aligned scheduling is feasible mainly in fixed‐shift systems (Stimpfel and Aiken 2013) and offers limited benefit in the rapidly rotating schedules common in Korean hospitals, where forward‐rotating patterns and adequate recovery may be more practical (Qtait et al. 2025). At the unit level, although direct evidence linking unit culture specifically to medication errors is limited, a nonpunitive culture in which fatigue and near‐misses can be reported without stigma may support early identification of at‐risk nurses and enable more complete reporting. Sleep and fatigue education alone has shown only limited, short‐term benefit and is best embedded in multicomponent programs that include CBT‐I; whether such programs reduce medication errors in perianesthesia settings requires prospective evaluation. Finally, because generic wellness programs show mixed effects, organizational investment in adequate staffing, on‐site rest facilities, and structured recovery represents a further organizational‐level approach whose effects on medication errors require contextual implementation and prospective evaluation (Pickup et al. 2025).

### 4.2 Limitations

This study addresses a largely overlooked population; however, it has several limitations. First, the descriptive correlational, cross‐sectional design supports associations rather than causality. Because reference periods differed across measures (insomnia, 2 weeks; fatigue, 1 month; overtime, 1 week; self‐reported errors, 3 months), exposures and outcomes were not temporally aligned: an error occurring weeks before data collection would not have been influenced by overtime reported for the most recent week. Prospective designs with shorter, aligned reference periods or repeated measures would better capture these temporal relationships.

Second, all measures were self‐reported and subject to response and recall bias, particularly for a sensitive outcome assessed over 3 months. Critically, nurses’ memory, awareness, and reporting of errors may be affected by fatigue, insomnia, and overtime, raising the possibility of differential misclassification of the outcome by exposure status. The confidential format, in which survey responses were not linked to identifying information, and the instruction to report errors regardless of formal reporting were intended to mitigate this limitation. Future work could triangulate self‐report with administrative incident records.

Third, overtime was self‐reported rather than drawn from work records, and the 1‐week measure is a snapshot that may not reflect typical exposure, an issue compounded by the modest sample. The error instrument captured frequency but not severity (errors reaching the patient with vs. without harm) and did not capture near‐misses; consequently, we cannot exclude an association between overtime and medication errors intercepted before reaching the patient. Allowing multiple error types per incident may also have inflated counts. Future studies should use work‐hour records and severity frameworks such as the National Coordinating Council for Medication Error Reporting and Prevention (NCC MERP) index.

Finally, the sample of 213 exceeded the requirement for effects of the magnitude observed (77 for IRR = 1.65) but fell short of the most conservative a priori estimate (230 for IRR = 1.35); therefore, smaller associations may have gone undetected. Moreover, the sample comprised predominantly nurses with 1–5 years of experience (67.1%), likely reflecting both the young Korean nursing workforce and the online and snowball recruitment, which may overrepresent digitally engaged nurses; the 3‐day survey period may have further limited eligible participants. Although duplicate participation was screened using the incentive contact list, undetected duplication cannot be entirely excluded under open online and snowball recruitment. With no national registry of Korean perianesthesia nurses, representativeness could not be formally assessed; larger multisite studies are needed for external validity.

## 5 Conclusion

This study investigated the prevalence of insomnia, fatigue, and overtime work among Korean perianesthesia nurses and their associations with the frequency of self‐reported medication errors. The findings revealed that clinically significant insomnia and fatigue were significantly associated with an increased summed self‐reported medication‐error‐type frequency score, whereas overtime work, though highly prevalent, did not show a significant association in the regression model. The most commonly reported error types were omission errors, wrong‐time administration, and improper dosing, suggesting areas that may warrant attention in high‐risk medication processes. These findings should be interpreted in consideration of the cross‐sectional design of the study, which precludes causal inference, and the reliance on self‐report, which may be subject to recall and reporting biases.

Given the critical role of perianesthesia nurses in ensuring medication safety in time‐sensitive and high‐pressure environments, our findings underscore the importance of addressing sleep health and fatigue as key components of patient safety. Pending confirmation in prospective studies, nurse managers and policymakers may consider interventions that address insomnia and fatigue, support sleep hygiene, and foster a culture of safety and openness around fatigue‐related risks.

## Author Contributions

Study design: YK, AM. Data collection: YK, AM. Data analysis: YK, AM, CP. Study supervision: AM. Manuscript writing: YK, EYC, WB, AM. Critical revisions for important intellectual content: EYC, WB, CP.

## Conflicts of Interest

The authors declare no actual or potential conflicts of interest.

## Funding Information

This work was supported by the National Research Foundation of Korea (NRF) grant funded by the Korean government (MSIT) (no. RS‐2025‐00521630).

## Supporting information




**Supporting File 1**: inr70233‐sup‐0001‐SuppMat.docx

## Data Availability

The data that support the findings of this study are available from the corresponding author upon reasonable request.
