# Supplementary material for: Associations of Insomnia, Fatigue, and Overtime Work With Self‐Reported Medication Errors Among Perianesthesia Nurses: A Cross‐Sectional Study
Source: Int Nurs Rev. 2026 Sep 6;73(3):e70233. doi: 10.1111/inr.70233 (PMC13547756; doi:10.1111/inr.70233)
Supplement: Supplementary file 1 [file INR-73-0-s001.docx]

**Supplementary Table 1.** Frequency of Individual Self-Reported Medication Error Types According to Insomnia, Fatigue, and Overtime Work Status (N = 213)

| Individual medication error types | Insomnia | | Fatigue | | Overtime work | |
| --- | --- | --- | --- | --- | --- | --- |
|  | z (p) | q | z (p) | q | z (p) | q |
| Omission error | −4.235 (<.001) | <.001 | −3.576 (<.001) | .002 | −2.734 (.006) | .017 |
| Wrong time | −2.204 (.028) | .065 | −2.683 (.007) | .019 | −1.787 (.074) | .121 |
| Improper dose/quantity | −4.769 (<.001) | <.001 | −4.502 (<.001) | <.001 | −2.944 (.003) | .011 |
| Prescribing error | −4.378 (<.001) | <.001 | −2.897 (.004) | .011 | −1.807 (.071) | .121 |
| Extra dose | −3.071 (.002) | .009 | −3.084 (.002) | .009 | −1.844 (.065) | .117 |
| Others | −1.086 (.278) | .349 | −1.847 (.065) | .117 | −1.078 (.281) | .349 |
| Wrong drug preparation | −2.102 (.036) | .075 | −2.958 (.003) | .011 | −2.187 (.029) | .065 |
| Unauthorized drug | −4.324 (<.001) | <.001 | −3.530 (.001) | .002 | −2.035 (.041) | .084 |
| Wrong administration technique | −1.532 (.126) | .196 | −1.455 (.146) | .218 | −1.435 (.151) | .218 |
| Wrong patient | −1.361 (.174) | .240 | 0.052 (.959) | .981 | 0.173 (.862) | .970 |
| Wrong route | 0.094 (.925) | .981 | 0.036 (.972) | .981 | 0.483 (.629) | .742 |
| Wrong dosage form | −1.251 (.211) | .281 | 0.024 (.981) | .981 | 0.469 (.639) | .742 |

*Note.* Mann–Whitney U tests were performed for all comparisons. Group comparisons were evaluated as follows: insomnia (clinically significant vs. absence), fatigue (fatigued vs. non-fatigued, defined by binary CFQ scoring ≥ 4), and overtime work (yes vs. no). The 36 item-level comparisons were treated as exploratory; q = Benjamini–Hochberg false discovery rate-adjusted q-value (q = .05) across the 36 tests.

**Supplementary Table 2.** Full Negative Binomial Regression Model for the Summed Self-Reported Medication-Error-Type Frequency Score (N = 213)

| Variables | β | IRR | 95% CI | | *p* |
| --- | --- | --- | --- | --- | --- |
|  |  |  | LLCI | ULCI |  |
| Insomnia |  |  |  |  |  |
| Clinically significant insomnia | 0.507 | 1.66 | 1.06 | 2.60 | .027 |
| Fatigue |  |  |  |  |  |
| Fatigued | 0.497 | 1.64 | 1.04 | 2.60 | .034 |
| Overtime work |  |  |  |  |  |
| Yes | 0.416 | 1.52 | 0.91 | 2.51 | .107 |
| Age (years) | −0.052 | 0.95 | 0.89 | 1.01 | .109 |
| Sex |  |  |  |  |  |
| Female | 0.539 | 1.71 | 0.78 | 3.77 | .179 |
| Marital status |  |  |  |  |  |
| Married | −0.397 | 0.67 | 0.31 | 1.44 | .307 |
| Children |  |  |  |  |  |
| Yes | 0.250 | 1.28 | 0.53 | 3.12 | .581 |
| Educational level |  |  |  |  |  |
| Bachelor's degree | 0.341 | 1.41 | 0.75 | 2.65 | .292 |
| Master's degree or higher | 0.899 | 2.46 | 0.84 | 7.21 | .102 |
| Perianesthesia experience (months) | −0.000 | 1.00 | 0.99 | 1.01 | .912 |
| Type of shift work |  |  |  |  |  |
| Rotating shift | 0.993 | 2.70 | 1.56 | 4.67 | <.001 |
| Scope of practice |  |  |  |  |  |
| Anesthesia and recovery care | −0.760 | 0.47 | 0.31 | 0.70 | <.001 |
| Number of beds |  |  |  |  |  |
| 500–999 | 0.260 | 1.30 | 0.84 | 1.99 | .236 |
| 300–499 | 0.088 | 1.09 | 0.63 | 1.89 | .753 |
| <300 | −0.065 | 0.94 | 0.44 | 2.02 | .868 |
| Commuting time |  |  |  |  |  |
| 31–60 min | −0.042 | 0.96 | 0.64 | 1.44 | .838 |
| ≥61 min | 0.268 | 1.31 | 0.74 | 2.31 | .358 |
| Number of night shifts in the last month | −0.090 | 0.91 | 0.83 | 1.01 | .078 |
| Experience of presenteeism |  |  |  |  |  |
| Yes | −0.013 | 0.99 | 0.66 | 1.47 | .950 |
| Caffeine intake frequency |  |  |  |  |  |
| 1–2 cups per month | 0.135 | 1.15 | 0.17 | 7.84 | .890 |
| 1–2 cups per week | 0.623 | 1.86 | 0.83 | 4.18 | .130 |
| 1–2 cups per day | 0.789 | 2.20 | 1.12 | 4.33 | .022 |
| ≥3 cups per day | 0.630 | 1.88 | 0.84 | 4.20 | .126 |
| Alcohol intake per consumption | 0.010 | 1.01 | 0.88 | 1.15 | .886 |

*Note.* IRR = incidence rate ratio, representing the relative expected value of the summed self-reported medication-error-type frequency score rather than the incidence rate of distinct medication-error events; CI = confidence interval; LLCI = lower limit of 95% confidence interval; ULCI = upper limit of 95% confidence interval; CFQ = Chalder Fatigue Questionnaire. Reference groups for independent variables: Insomnia = absence of insomnia; Fatigue = non-fatigued (fatigued defined by binary CFQ scoring ≥4); Overtime work in the last week = no; Sex = male; Marital status = unmarried; Children = no; Educational level = associate degree; Type of shift work = fixed shift; Scope of practice = anesthesia nursing; Number of beds = ≥1,000 beds; Commuting time = ≤30 min; Experience of presenteeism = no; Caffeine intake frequency = none. Overall model: LR χ²(24) = 81.27, *p* < .001; pseudo R² = .084. Dispersion parameter α = 0.97 (95% CI 0.71–1.32); likelihood-ratio test of α = 0: χ̄²(01) = 253.83, *p* < .001.

**Supplementary Table 3.** Sensitivity Analysis: Binary Logistic Regression Comparing Nurses Reporting at Least One Medication-Error Type with those Reporting None (N = 213)

| Variables | OR | 95% CI | | *p* |
| --- | --- | --- | --- | --- |
|  |  | LLCI | ULCI |  |
| Insomnia |  |  |  |  |
| Clinically significant insomnia | 2.58 | 0.86 | 7.73 | .091 |
| Fatigue |  |  |  |  |
| Fatigued | 2.49 | 1.07 | 5.80 | .035 |
| Overtime work |  |  |  |  |
| Yes | 2.05 | 0.81 | 5.17 | .127 |
| Age (years) | 0.87 | 0.78 | 0.98 | .022 |
| Sex |  |  |  |  |
| Female | 1.97 | 0.47 | 8.22 | .351 |
| Marital status |  |  |  |  |
| Married | 0.44 | 0.12 | 1.69 | .234 |
| Children |  |  |  |  |
| Yes | 1.09 | 0.23 | 5.13 | .912 |
| Educational level |  |  |  |  |
| Bachelor's degree | 2.79 | 0.69 | 11.30 | .150 |
| Master's degree or higher | 8.66 | 1.01 | 74.08 | .049 |
| Perianesthesia experience (months) | 1.00 | 0.99 | 1.01 | .914 |
| Type of shift work |  |  |  |  |
| Rotating shift | 5.24 | 1.78 | 15.39 | .003 |
| Scope of practice |  |  |  |  |
| Anesthesia and recovery care | 0.26 | 0.11 | 0.62 | .002 |
| Number of beds |  |  |  |  |
| 500–999 | 1.68 | 0.66 | 4.32 | .280 |
| 300–499 | 1.45 | 0.43 | 4.85 | .545 |
| <300 | 1.20 | 0.30 | 4.86 | .797 |
| Commuting time |  |  |  |  |
| 31–60 min | 0.62 | 0.26 | 1.49 | .283 |
| ≥61 min | 1.24 | 0.37 | 4.17 | .725 |
| Number of night shifts in the last month | 0.83 | 0.69 | 1.01 | .057 |
| Experience of presenteeism |  |  |  |  |
| Yes | 1.93 | 0.87 | 4.28 | .104 |
| Caffeine intake frequency |  |  |  |  |
| 1–2 cups per month | 1.06 | 0.00 | 680.42 | .985 |
| 1–2 cups per week | 3.43 | 0.80 | 14.75 | .098 |
| 1–2 cups per day | 4.12 | 1.25 | 13.62 | .020 |
| ≥3 cups per day | 3.23 | 0.74 | 14.11 | .119 |
| Alcohol intake per consumption | 0.91 | 0.70 | 1.19 | .486 |

*Note.* OR = odds ratio; CI = confidence interval; LLCI = lower limit of 95% confidence interval; ULCI = upper limit of 95% confidence interval; CFQ = Chalder Fatigue Questionnaire. Reference groups for independent variables: Insomnia = absence of insomnia; Fatigue = non-fatigued (fatigued defined by binary CFQ scoring ≥4); Overtime work in the last week = no; Sex = male; Marital status = unmarried; Children = no; Educational level = associate degree; Type of shift work = fixed shift; Scope of practice = anesthesia nursing; Number of beds = ≥1,000 beds; Commuting time = ≤30 min; Experience of presenteeism = no; Caffeine intake frequency = none. The model used the same covariates as the principal negative binomial model. Overall model: LR χ²(24) = 86.49, *p* < .001; pseudo R² = .316. The wide confidence interval for the caffeine intake of 1–2 cups per month reflects the small number of nurses in that category (n = 2).
